# Efficacy of Bushen Huatan Decoction combined with Baduanjin in the treatment of polycystic ovary syndrome with insulin resistance (IR-PCOS), kidney deficiency and phlegm dampness: study protocol for a randomized controlled trial

**DOI:** 10.1186/s13063-021-05770-z

**Published:** 2021-11-07

**Authors:** Haiqing Qian, Wenting Xu, Lijuan Cui, Rong Wang, Jiahui Wang, Mengyu Tang, Minggang Wei, Lihong Wang

**Affiliations:** 1 Department of Reproduction, Zhangjiagang TCM Hospital Affiliated to Nanjing University of Chinese Medicine, Zhangjiagang, Suzhou, Jiangsu China; 2 Department of Pathology, Zhangjiagang TCM Hospital Affiliated to Nanjing University of Chinese Medicine, Zhangjiagang, Suzhou, Jiangsu China; 3 Department of Laboratory Medicine, Zhangjiagang TCM Hospital Affiliated to Nanjing University of Chinese Medicine, Zhangjiagang, Suzhou, Jiangsu China; 4 The First Affiliated Hospital of Soochow University, Suzhou, Jiangsu China

**Keywords:** Polycystic ovary syndrome, Insulin resistance, Bushen Huatan Decoction, Baduanjin

## Abstract

**Background:**

Polycystic ovary syndrome (PCOS) is a common reproductive endocrine disease in women. Insulin resistance (IR) has emerged as a central contributor to the pathogenesis of this disease. According to traditional Chinese medicine (TCM), kidney deficiency is the main syndrome of PCOS. The deficiency of the kidney cannot vaporize water-dampness, and the retention of water-dampness accumulates into phlegm dampness stagnation, resulting in visceral dysfunction and metabolic disorder. TCM involving syndrome differentiation and treatment is widely used to adjust women’s menstrual cycles. Our patented formula Bushen Huatan Decoction (BSHTD) has been proven to be effective in the clinical treatment of IR-PCOS. Baduanjin also plays an important role in improving metabolic syndrome through lifestyle intervention. This study investigates the clinical efficacy of Bushen Huatan Decoction combined with Baduanjin in IR-PCOS, to form a specific TCM-behaviour intervention plan in the treatment of IR-PCOS.

**Methods/design:**

This is a randomized controlled trial involving 190 participants diagnosed with IR-PCOS. All participants will be randomly allocated into 5 groups: group A will receive metformin; group B, BSHTD; group C, Baduanjin; group D, BSHTD combined with metformin; and group E, BSHTD combined with Baduanjin. One course of treatment lasts 3 months, a total of two courses. The primary outcomes are changes in the homeostatic model assessment of insulin resistance (HOMA-IR) and improvements in the oral glucose tolerance test (OGTT) and insulin-releasing test (INS). The secondary outcomes are improvements in the menstrual cycle, ovulation rate, clinical pregnancy rate, basic serum sex hormone levels, free androgen index (FAI), Ferriman-Gallwey scores, body mass index (BMI) and TCM syndrome scores. The related observation indexes will be collected at baseline, during the process of treatment and at the 6-month follow-up. Simultaneously, close monitoring of possible adverse events will be performed throughout the trial process.

**Discussion:**

This trial will investigate the efficacy of the comprehensive intervention program of Bushen Huatan Decoction combined with Baduanjin on the adjustment of the menstrual cycle, improvement of insulin resistance and correction of glucose metabolism disorder in IR-PCOS patients. It is expected to form an alternative treatment of TCM-behaviour intervention therapy for IR-PCOS and promote the Chinese fitness Qigong Baduanjin in the application of lifestyle diseases.

**Trial registration:**

Chinese Clinical Trial Registry ChiCTR2100043415. Registered on 15 February 2021.

**Supplementary Information:**

The online version contains supplementary material available at 10.1186/s13063-021-05770-z.

## Background

Polycystic ovary syndrome (PCOS) is one of the most common reproductive endocrine and metabolic disorders in modern women and affects approximately 4% to 8% of women of childbearing age worldwide [1]. With the acceleration of life rhythm and transformation of lifestyle, the incidence of this disease is increasing year by year and has become one of the main causes of female ovulation disorder infertility [2]. The clinical manifestations of PCOS are highly heterogeneous and complex; doctor’s office visits usually occur for patients’ oligomenorrhoea, amenorrhoea, infertility, abnormal uterine bleeding, obesity, hirsutism, acne, etc.

At present, the pathogenesis of PCOS is not completely clear; there is growing evidence that PCOS may be a complex polygenic disease and under the influence of epigenetics and the environment, accompanied by a chronic low-grade pro-inflammatory state [3]. Insulin resistance (IR) is the central link in the occurrence of this disease [4], and the mechanism is related to abnormalities in the insulin receptor signal transduction pathway [5]. Obesity-induced IR and hyperandrogenemia are independent factors for the evolution of PCOS. If the patients’ metabolic imbalance and endocrine disorders are not corrected in a reasonable length of time, long term complications associated with PCOS including abnormal metabolism of glucose and lipids, cardiovascular and cerebrovascular diseases, oestrogen-dependent tumours (such as breast cancer and endometrial cancer) and mental illness can seriously affect a woman’s quality of life and threaten her health [6].

According to TCM, the pathogenesis of PCOS is based on deficiency of spleen and kidney, with phlegm dampness and blood stasis as the appearance, especially kidney deficiency and phlegm dampness as the most common pathological type of PCOS. Kidney deficiency includes insufficiency of kidney-essence, kidney-Qi, kidney-Yin, kidney-Yang and other aspects, which can lead to insufficiency of generating essence and transforming blood, thus affecting the cycle of menstruation and embryo breeding. Patients with IR-PCOS tend to have a fat-greasy diet, coupled with a lack of good lifestyle and exercise, resulting in phlegm dampness congestion and hindrance of functional activities of Qi, so that disharmony of Chong and Ren channels and amenorrhoea. In addition, clinical symptoms such as follicular growth restriction, ovulation dysfunction and abnormal metabolism of glucose and lipid caused by kidney deficiency and phlegm dampness are closely related to the pathological link of insulin resistance.

The main treatment strategies for PCOS are correcting insulin resistance, improving hyperandrogenemia and its signs, and promoting ovulation and fertility [7]. A growing number of studies and guidelines recommend lifestyle adjustment as the first-line treatment for PCOS. Lifestyle intervention is mainly reflected by alterations in diet, exercise, sleep, emotion and so on. As a kind of gradual approach, exercise intervention should be carried out scientifically and rationally. Baduanjin is a form of traditional Chinese exercise Qigong that follows the theoretical system of TCM. Its movements consist of eight separate parts, and it leads to adjustments to overall physical fitness through the movement of the muscles and joints, the control of breathing, and the concentration of the mind [8], so as to maintain the harmony between Qi and blood, keep Yin and Yang in balance. It plays an active role in the treatment of chronic diseases and metabolic syndrome [9]. Traditional Chinese medicine has been widely used in the treatment of metabolic syndrome; our previous basic study confirmed that Bushen Huatan Decoction can activate the PI3K/AKT pathway [10] and increase the expression levels of insulin receptor (INSR) and IRS-1 protein in adipose tissue of IR-PCOS model rats, thus improving glucose and lipid metabolism and insulin resistance in rats. In addition, our clinical process has proven that Bushen Huatan Decoction can obviously improve insulin sensitivity, reduce androgen levels and correct reproductive cycles.

Therefore, we will combine Bushen Huatan Decoction with Baduanjin to intervene in IR-PCOS of kidney deficiency and phlegm dampness in this trial, evaluate its clinical efficacy and examine the advantages of traditional Chinese medicine and Chinese Qigong exercise in the treatment of this disease to form a specific combined program of TCM-behaviour intervention for IR-PCOS of kidney deficiency and phlegm dampness, to maintain patients’ health and improve their quality of life.

## Methods/design

### Study design

This is a factorial-design randomized controlled clinical trial. Multi-channel intervention of IR-PCOS will be evaluated simultaneously by treating the different combinations of metformin, BSHTD and Baduanjin exercise, so as to find the best intervention scheme for the treatment of this disease. The research protocol is compliant with the Consolidated Standards of Reporting Trials (CONSORT) guidelines [11] as well as the Standard Protocol Items: Recommendations for Interventional Trials (SPIRIT) statement [12]. The SPIRIT checklist is presented in additional file 1.

This study has been approved by the ethics committee of Zhangjiagang TCM Hospital Affiliated to Nanjing University of Chinese Medicine. The patients will be recruited from the Department of Reproduction of Zhangjiagang TCM Hospital Affiliated to Nanjing University of Chinese Medicine. Informed consent will be obtained from each participant before the study procedure is performed according to good clinical practice. This trial has been registered at the Chinese Clinical Trial Registry (ChiCTR2100043415) and conducted in accordance with the Declaration of Helsinki.

### Participants

A total of 190 patients who met the inclusion criteria without any exclusion criteria will be recruited in this study through the physicians from the department of reproduction of Zhangjiagang TCM Hospital Affiliated to Nanjing University of Chinese Medicine. All participants will be provided with the background information, purpose, possible benefits and harms of this study before signing informed consent. The written documents related to informed consent will be signed face-to-face by physicians and patients during outpatient diagnosis and treatment and will be kept by a member of the project team who will never be involved in the clinical data collection throughout the whole process. The recruitment plan will begin in March 2021 and be completed in March 2022.

#### Inclusion criteria

1. Chinese women aged from 18 to 35 years old.
2. BMI [13] over 25.0 kg/m^2^.
3. Confirmed diagnosis of PCOS according to the 2003 Rotterdam criteria [14]: (a) irregular menstruation cycles (a periodic interval > 35 days or < 8 cycles in a year) or amenorrhoea (a periodic interval > 90 days) or abnormal uterine bleeding; (b) clinical manifestations of hyperandrogenism (hirsutism in mainland China is taken as Ferriman-Gallwey score ≥5 [15]) and/or hyperandrogenemia [free androgen index (FAI)=total testosterone (nmol/L)/SHBG (nmol/L)×100 > 4.5 [16]]; and (c) transvaginal ultrasonography showing PCOM: the number of follicles with a diameter of 2–9 mm in one or both ovaries ≥12, and/or ovarian volume ≥10 ml [ovarian volume calculation: 0.5 × long diameter (cm) × diameter (cm) × anteroposterior diameter (cm)]. Among all the above, (a) is a necessary condition, and matching one of (b) or (c) represents suspected PCOS, which then requires ruling out other factors and diseases that may cause hyperandrogenism and ovulation dysfunction.
4. In line with the diagnosis criteria of TCM syndrome differentiation: kidney deficiency and phlegm dampness.
5. Confirming the diagnosis of insulin resistance: the criteria of IR are defined according to the homeostatic model assessment of insulin resistance (HOMA-IR) [fasting plasma glucose (mmol/l) × fasting serum insulin (uIU/ml)/22.5]. A value ≥2.29 [17] is considered to be indicative of IR.
6. No history of using hormone drugs or drugs that affect glucose metabolism within the 3 months prior to treatment.
7. Volunteer to participate in this trial and give informed consent.

#### Exclusion criteria

1. Patients with any other endocrine diseases related to this disease (such as Cushing’s syndrome, congenital adrenal hyperplasia, 21-hydroxylase deficiency, hyperprolactinemia, uncorrected thyroid disease, type 1 or type 2 diabetes mellitus, etc. ).
2. Patients with congenital malformations or defects of the reproductive system, organic lesions of the reproductive system (such as uterine fibroids and ovarian cysts).
3. Patients with reproductive malignancies (such as endometrial cancer and cervical cancer).
4. Patients with genital inflammation, genital tuberculosis, and pelvic inflammatory disease.
5. One of the spouses has a sexually transmitted disease such as syphilis and AIDS.
6. Patients with serious primary diseases of the cardiovascular, cerebrovascular, liver, kidney and hematopoietic systems or psychoses.
7. Patients with chromosomal abnormalities, pregnancy or lactation.
8. Interventions of any other medication treatments, including hormone drugs, drugs that will affect glucose metabolism, Chinese herbal prescriptions or acupuncture in the previous 3 months.
9. Patients allergic to the drugs used in this study.
10. Patients in other clinical trials at the same time.

### Interventions

After baseline measurements, participants satisfying the inclusion criteria will be randomly divided into one of the following 5 clinical groups:

1. Group A: metformin at a dose of 500 mg per meal, 3 times per day, orally for 2 courses.
2. Group B: BSHTD at a total dose of 400 ml per day, divided into 2 oral, doses for a total of 2 courses.
3. Group C: Baduanjin exercise for 30 min per day, for a total of 2 courses.
4. Group D: BSHTD combined with metformin, orally for 2 courses.
5. Group E: BSHTD combined with Baduanjin exercise, for a total of 2 courses.

#### Group A: Metformin

For the purpose of reducing symptoms of gastrointestinal discomfort caused by metformin, the daily oral dose will be gradually increased from 500 mg during the first week, to 1000 mg during the second week, and to 1500 mg during the third week. From the fourth week onwards, the daily oral dose will be maintained at 500 mg per meal, 3 times per day (orally along with breakfast, lunch and dinner). One course of treatment is three months long, and patients will be given a total of two courses.

#### Group B: Bushen Huatan Decoction

The Bushen Huatan Decoction is composed of 12 g of *Cuscuta chinensis Lam*, 12 g of *Astragali Radix*, 10 g of *Atractylodis Rhizoma*, 12 g of *Cyperi Rhizoma*, 10 g of *Poria*, 9 g of *Pinelliae Rhizoma*, and 6 g of *Citri Reticulatae Pericarpium*. The patients will take a total dose of 400 ml, divided into 2 oral doses per day (1 h after breakfast and 1 h after dinner), and will stop taking it during menstruation. In the course of the trial, the TCM doctor will add or subtract the herbal medicine according to the patients’ systemic and local concomitant symptoms. One course of treatment is three months long, and patients will be given a total of two courses. If a pregnancy test is positive during the course, the Bushen Huatan Decoction will be changed to kidney-nourishing and foetal soothing prescriptions to prevent miscarriage.

#### Group C: Baduanjin exercise

The Baduanjin exercise newly promulgated by the Ministry of Sport of China in 2003 will be adopted in this trial. We will guide the patients to learn Baduanjin exercise through 8 courses and ensure that all participants can master standard movements. After that, the patients will consciously perform these exercises at home once a day, for 30 min each session, and will stop during menstruation. One course of treatment is three months long, and patients will be given a total of two courses. We will provide free training videos and daily training registration cards to urge and monitor practice attendance.

#### Group D: Bushen Huatan Decoction combined with metformin

The patients will take a total dose of 400 ml of Bushen Huatan Decoction, divided into 2 oral doses per day (1 h after breakfast and 1 h after lunch), and stop the intervention during menstruation, together with the daily dose of metformin (the regimen is the same as that of group A).

#### Group E: Bushen Huatan decoction combined with Baduanjin exercise

The daily oral solution of Bushen Huatan Decoction is similar to that of group B; in addition, the participants in this group are required to practice Baduanjin exercise during the stage of the trial (the specific exercise program is similar to that of group C). All interventions will be stopped during menstruation. One course of treatment is three months long, and patients will be given a total of two courses.

#### Follow-up

To assess the accuracy of diagnosis, effectiveness and safety of treatment in individual interventions, a follow-up via face-to-face interview, telephone, WeChat, text message, or e-mail will be conducted 6 months after the final treatment. During this stage, none of the subjects will receive any other therapy other than routine cervical care. The case observers will investigate the situation of all participants, mainly including menstrual cycle, TCM syndromes and signs, the appearance of hyperandrogenism (body weight, excessive hairiness, acne, acanthosis nigricans) and pregnancy. Participants are welcome to inform the assessors of their clinical symptoms at additional times throughout the experimental period.

#### Patient and public involvement

This trial was designed by investigators in accordance with previous clinical experience and pertinent literature. No patients were involved in the design of this study. All participants will be guided to learn Baduanjin exercise through 8 educational courses. We will also give general information about IR-PCOS in these courses and encourage the participants to ask questions and give us feedback to ensure the smooth progress of this trial. The cost of drug interventions and laboratory tests, and the registration fees during follow-up will be covered by study fundings so it will not be a significant burden and will be in line with participants preferences. We will also send out a summary of their own clinical data and the study results to all participants at the completion of the trial.

### Study-specific visits and procedures

**Fig. 1:**
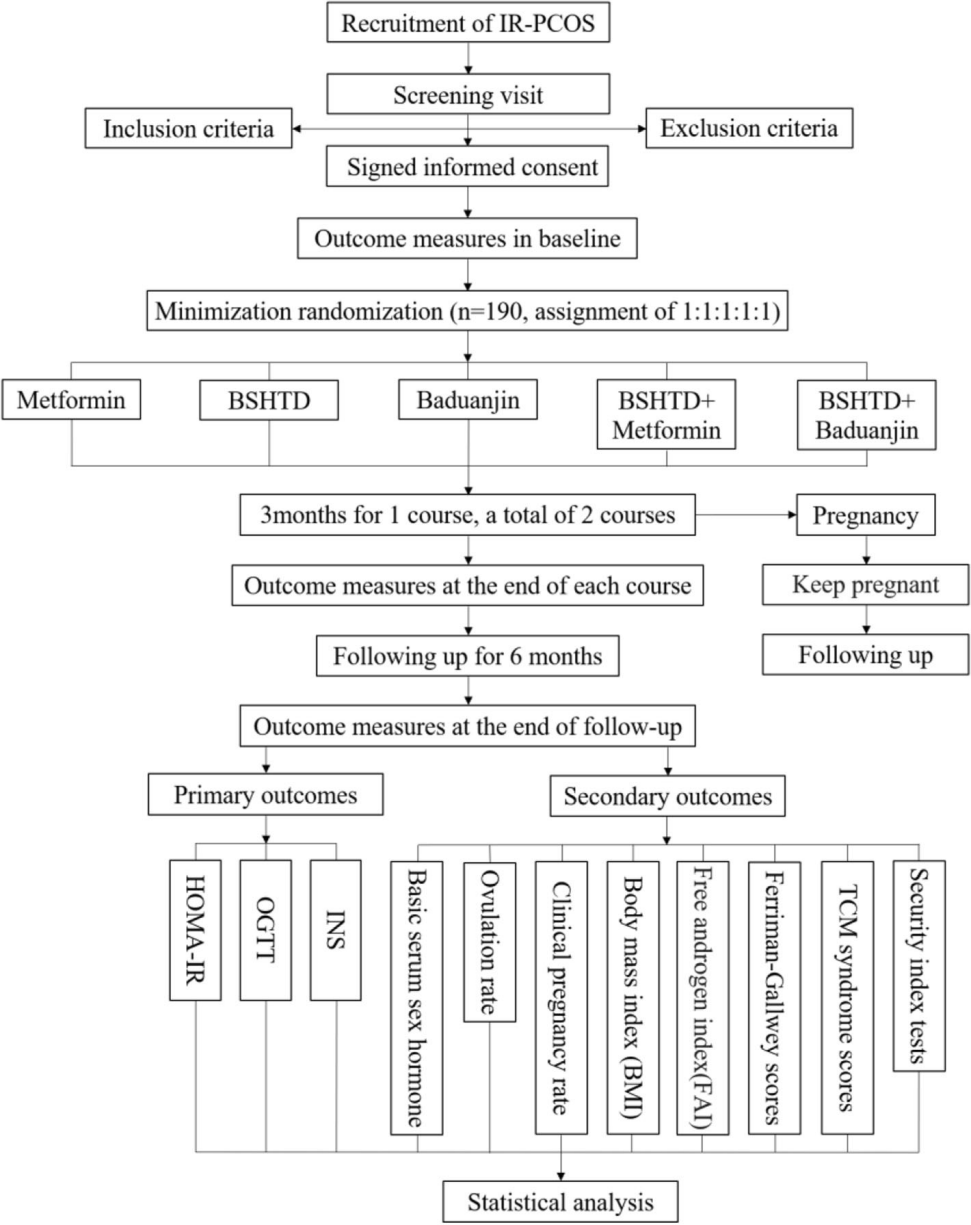
Flow chart of the trial

All participants will receive 2 courses of treatment lasting for 6 months and will be followed up for 6 months. All participants will attend a total of 6 visits: screening visit, baseline visit, first course visit, end-of-treatment visit, and two follow-up visits. Simultaneously, close monitoring of possible adverse events during the trial process will be performed. A flowchart of this trial is depicted in Fig. 1. The schedule of trial enrolment, intervention and assessment is expounded in Table 1.

**Table 1:**
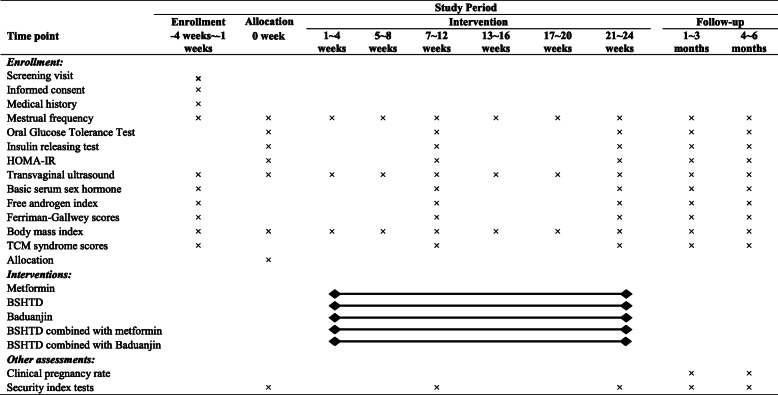
Schedule of enrolment, intervention and assessment

### Outcome measurements

#### Primary outcomes

The primary outcomes are changes in HOMA-IR (calculated according to the fasting plasma glucose and serum insulin) and improvements in the oral glucose tolerance test (OGTT) and insulin-releasing test (INS).

#### Secondary outcomes

The secondary outcomes will include the levels of basic serum sex hormones [including luteinizing hormone (LH), follicle-stimulating hormone (FSH), oestradiol (E_2_), testosterone (T), progesterone (P), prolactin (PRL), dehydroepiandrosterone (DHEAS), and sex hormone binding globulin (SHBG)], the ovulation rate in each menstrual cycle, the clinical pregnancy rate, BMI, FAI, Ferriman-Gallwey scores, TCM syndrome scores and security index tests (including routine blood examination, routine urine and stool tests, hepatic and renal function, and ECG examination).

All the participants will be instructed to return to visit the doctor every week. They will be contacted by phone if they miss a scheduled appointment. All clinical progress, adverse events and additional medication use will be recorded at every visit.

### Sample size calculation

The sample size calculated by Power Analysis & Sample Size (NCSS-PASS 11.0) was assessed based on HOMA-IR, which is the most significant outcome in this trial. In our pilot study (according to the research results of Dr. Li-hong Wang’s doctoral thesis) evaluating the effect of metformin and BSHTD on HOMA-IR in patients with IR-PCOS, the results showed that HOMA-IR decreased from 2.11±0.30 to 1.71±0.26 in the metformin treatment group and the difference before and after treatment was 0.40±0.24, while HOMA-IR of Bushen Huatan Decoction combined with metformin treatment group decreased from 2.09±0.30 to 1.52±0.26 and the difference before and after treatment was 0.57±0.27. Therefore, we used the minimum sample size required to evaluate effectiveness [18]. We anticipate that after two courses of treatment, HOMA-IR of Bushen Huatan Decoction combined with metformin group will decrease by 30%, and HOMA-IR of single-use of metformin group will decrease by 20%. Therefore, the difference is 10%,with two-sided α assigned to be 5% and *β* 20% at the upper limit, respectively, at which the power is 90% and the rate of attrition estimated to be 20%. In a one-way ANOVA study, sample sizes of 30, 30, 30, 30, and 30 were obtained for the 5 groups whose means are to be compared. A total sample size of 150 subjects achieves 90% power to detect differences among the means versus the alternative of equal means using an F test with a 5% significance level. By enrolling 38 participants in each group, we allow the rate of attrition to be estimated to be 20%.

### Randomization and allocation concealment

The eligible participants will be allocated into 5 groups at a ratio of 1:1:1:1:1. Researchers of the Department of Reproduction of Zhangjiagang TCM Hospital Affiliated to Nanjing University of Chinese Medicine will use a random number generator of the Statistical Package for Social Sciences (SPSS) version 25.0 to carry out the randomization process. The random sequences will be put into several opaque paper envelopes, numbered sequentially and then distributed to clinical researchers and doctors [19]. When new patients are recruited in this trial, the opaque paper envelopes will be opened to decide their distribution; moreover, the researchers will arrange the corresponding interventions for the patients. All procedures above will be maintained and followed as appropriate.

### Blinding

This study is a single-blinded trial. Outcome assessors and people responsible for statistical analysis will be blinded to the randomization status.

### Data collection and management

Documentations such as paper case report forms (CRFs) will be applied to record relevant information (personal identity information, clinical data, etc.) of each participant. All medical information will be accurately recorded in the form of a double check by two researchers of the project team when inputted from the CRFs, monitored and validated by the data coordinating committee statisticians, and kept strictly confidential in a web-based electronic database, ResMan (www.medreaman.org), that needs special permissions. Information in the database and all original paper materials will be stored by a member of this trial who will never be involved in the clinical data collection throughout the whole process and the project team will ensure that the CRFs will be delivered securely to the Trial Office for data entry. All members of this study will keep the participants’ information confidential.

### Statistical analysis

Clinical data of all participants will be disposed in the final statistical analysis based on the intent-to-treat principle to determine the robustness of the evidence. The data processors will be unaware of the specific allocation situation. Basic indicators will be analysed to test the balance of each group at baseline. Comparisons will be processed based on changes from baseline to the end of the intervention and follow-up. The one-sample Kolmogorov-Smirnov test and graphical methods will be applied to test the normal distribution of continuous variables (such as the levels of basic serum sex hormones, HOMA-IR, ovulation rate, clinical pregnancy rate, BMI, FAI, Ferriman-Gallwey scores and TCM syndrome scores), which will be presented as the mean ± standard deviation if normally distributed and as the median with an interquartile range if not normally distributed. A paired *T* test or the Wilcoxon rank test will be used to compare variables within a group from baseline to the end of the intervention. Analysis of variance (ANOVA) with Bonferroni correction will be carried out to compare differences among groups of continuous variables consistent with a normal distribution. Continuous variables that do not meet the normal distribution will be processed by using the Kruskal-Wallis test followed by the Mann-Whitney *U* test. Categorical variables will be analysed by chi-square statistics when appropriate, and Fisher’s exact test will be used to compare discrete variables with fewer than 5 observations. All data analyses will be processed by the Statistical Package for Social Sciences (SPSS) version 25.0. A *P* value < 0.05 with a two-tailed test and mean difference with 95% confidence intervals will be considered statistically significant.

### Imputation procedure for missing data

We will report the reasons for intervention withdrawal in different randomized subgroups and conduct a qualitative analysis of the reasons. The impact of any missing data on the study results will be assessed by sensitivity analysis of the augmented data sets. Patients who drop out of the trial will be enrolled in the analysis of missing data using modern imputation methods.

### Quality assurance

A diverse study group including the principal investigator, recruiters, physicians, medical laboratory, ethics committee, drug supervision and administration department, data coordinating centre and patients will be established before the study, and all of the team members will be trained to understand the design, purpose, and basic information of the trial. In order to ensure that the procedure of the treatment is of high standard and delivered in accordance with the trial protocol, all physicians of the project team will have a certificate of TCM and at least one year of working experience. Before the beginning of the study, each member of the project team has already completed the study-specific theoretical course and undergone the study-specific practical skills training once a day for a month. All physicians have passed the theoretical and practical assessment.

### Ethics and dissemination

The protocol has been approved by the ethics committee of Zhangjiagang TCM Hospital Affiliated to Nanjing University of Chinese Medicine. This trial has been registered at the Chinese Clinical Trial Registry (ChiCTR2100043415) and conducted in accordance with the Declaration of Helsinki. The results of this trial will be disseminated through publication in peer-reviewed journals.

### Safety

The safety of BSHTD has been clinically confirmed. Baduanjin exercise is considered as the traditional Chinese fitness Qigong, and metformin is considered as the first-line treatment for correcting the insulin resistance of PCOS and is not expected to be associated with adverse events when administered in accordance with a normal daily dose. Nevertheless, all participants will be required to report potential adverse events at each trial point (baseline, during the process of treatment and at the 6 months follow-up). In case of unexpected serious adverse events (SAEs), the patient shall withdraw from the trial at any time and go to the corresponding departments of our hospital for free treatment, and the researchers will closely observe the patients until they recover. All adverse events occurring during the study period will be documented in CRFs. SAEs will be reported to the principal investigator, ethics committee and data coordinating centre within 24 h. The principal investigator is responsible for the management of security reports according to local guidelines. Once the SAEs occur, it will be immediately referred to the ethics committee for review. The ethics committee has the final say on the termination of the trial.

## Discussion

As far as we are aware, no previous investigation has discussed whether Baduanjin exercise has therapeutic potential in IR-PCOS. Our research is the first randomized controlled trial to compare the efficiency of a combination of TCM and behavioural therapy in the treatment of IR-PCOS. Each aspect of this trial was fully discussed by the research group, and this protocol was unanimously approved by the group. The assumptions of this trial are as follows: Bushen Huatan Decoction shows equivalent clinical effects as the single-use of metformin; the efficiency of Bushen Huatan Decoction combined with metformin is superior to that of a single-use of metformin; the efficiency of Bushen Huatan Decoction combined with Baduanjin exercise is superior to that of Bushen Huatan Decoction and Baduanjin exercise; and the efficiency of Bushen Huatan Decoction combined with Baduanjin is equal to that of Bushen Huatan Decoction combined with metformin.

From the perspective of TCM syndrome differentiation and treatment, kidney deficiency and phlegm dampness are mainly responsible for the pathogenesis of IR-PCOS, so the central focus of treatment should be on tonifying the kidney, invigorating the spleen and reducing phlegm, while paying attention to lifestyle modification in the course of treatment. It is worth noting that patients with IR-PCOS appear to have a higher risk of irregular diet and lifestyle. However, taking weight-loss drugs for a long time is not a sustainable solution for overweight patients; from this, we developed a comprehensive intervention program that combines TCM with Chinese fitness Qigong Baduanjin for the treatment of patients with IR-PCOS. On the one hand, a lifestyle-based intervention program can provide considerable benefits, such as body weight loss and improvements in physical signs of hyperandrogenism. On the other hand, the program can improve the psychosocial status of patients and provide a better quality of life. Additionally, a social network platform will be established to motivate and encourage the patients throughout the intervention and follow-up phases.

In summary, the aim of this trial is to conduct a pragmatic study that is expected to provide reliable evidence for using TCM-behaviour intervention therapy in future clinical practice in IR-PCOS and promote the application of Chinese fitness Qigong Baduanjin in the treatment of lifestyle diseases. We consider that this trial will contribute significant information for further recommendations regarding lifestyle interventions in females with IR-PCOS.

## Trial status

This trial was registered at the Chinese Clinical Trial Registry on February 15, 2021. Recruitment is anticipated to start in March 2021 and is expected to be finished by March 2022. The trial is predicted to be completed by January 2023, considering the 6 months follow-up time for collecting information. This is version 2.0 of the protocol, dated January 2021.

## Supplementary Information

**Additional file 1.** Standard Protocol Items: Recommendations for Interventional Trials (SPIRIT) 2013 Checklist: recommended items to address in a clinical trial protocol and related documents.

## Data Availability

Relevant information (personal identity information, clinical data, etc.) of each participant will be stored in a web-based electronic database ( e.g. ResMan, www.medreaman.org) that requires special permissions. The informed consent will solicit each participant whether they will allow all sponsors of this trial ( i.e. all authors of this paper ) to have access to the clinical data. The project team promises to take responsibility for the completeness and accuracy of the data in this study and to keep the information confidential. Clinical biological samples ( e.g. blood samples for fasting plasma glucose and serum insulin and oral glucose tolerance test blood samples) will be collected. If patients withdraw from the trial, their permission will be sought to use their clinical biological samples and clinical data. Relevant clinical biological samples will be laboratory analysed and stored by researchers from the Department of Laboratory Medicine of Zhangjiagang TCM Hospital Affiliated to Nanjing University of Chinese Medicine. As the trial is ongoing, the datasets generated or analysed during the current study are not publicly available. Upon the completion of the trial, supporting data for the study may be available to participants and publications upon reasonable request.

## Abbreviations

PCOS: Polycystic ovary syndrome
IR: Insulin resistance
HOMA-IR: Homeostatic model assessment of insulin resistance
TCM: Traditional Chinese medicine
BSHTD: Bushen Huatan decoction
OGTT: Oral glucose tolerance test
FAI: Free androgen index
INS: Insulin-releasing test
BMI: Body mass index
LH: Luteinizing hormone
FSH: Follicle-stimulating hormone
E_2_: Oestradiol
T: Testosterone
P: Progesterone
PRL: Prolactin
DHEAS: Dehydroepiandrosterone
SHBG: Sex hormone binding globulin
CRFs: Case report forms
ANOVA: Analysis of variance
SPSS: Statistical Package for Social Sciences
SAEs: Serious adverse events.
